# A Decade of Change in Peritoneal Dialysis in Brazil: Challenges and Perspectives in the Public Health System

**DOI:** 10.3390/healthcare13030337

**Published:** 2025-02-06

**Authors:** José A. Moura-Neto, Luís Gustavo Modelli de Andrade, Ana Flávia Moura, Constança Margarida Sampaio Cruz

**Affiliations:** 1 Department of Internal Medicine, Bahiana School of Medicine and Public Health, Salvador 40290-000, BA, Brazil; anaflaviamoura@bahiana.edu.br (A.F.M.); constancacruz@yahoo.com.br (C.M.S.C.); 2 Department of Internal Medicine, UNESP, Universidade Estadual Paulista, Botucatu 18618-681, SP, Brazil; gustavo.modelli@unesp.br

**Keywords:** peritoneal dialysis, renal replacement therapy, Brazil, health policy, public health systems, health disparities

## Abstract

**Background/Objectives:** The Brazilian Constitution defines health as a universal right and a State responsibility, with the Unified Public Health System (SUS) ensuring free access to comprehensive care, including renal replacement therapies (RRTs) such as dialysis and kidney transplantation. This study aimed to analyze trends in peritoneal dialysis (PD) usage within Brazil’s public health system over a 10-year period, focusing on geographic, demographic, and clinical changes. **Methods:** Using data from DATASUS and the Brazilian Society of Nephrology Dialysis Census, we analyzed PD usage and patient characteristics from 2014 to 2023. This methodology enabled an in-depth examination of shifts in RRT trends across regions and patient demographics. **Results:** PD usage declined from 6.5% in 2014 to 4.3% in 2023, with the steepest reductions observed in the North and Northeast regions. Usage increased in the Central-West region, while the Southeast and South experienced steady declines from 2016 to 2023. The proportion of centers offering PD decreased from 51.6% in 2014 to 37.9% in 2023. Over time, the average age of PD patients increased, as did the proportion of Brown/Black individuals receiving PD. Despite these shifts, patient serum levels of hemoglobin, parathyroid hormone, and phosphorus remained stable. **Conclusions:** This study highlights a relative decline in PD availability and use within Brazil’s public health system, with notable regional disparities. These findings underscore the urgent need for targeted policies to support PD infrastructure, funding, and training to ensure equitable access to RRT across the country.

## 1. Introduction

Peritoneal dialysis (PD) is a valuable modality of renal replacement therapy (RRT) for patients with end-stage kidney disease (ESKD) worldwide. However, its utilization varies significantly across regions, reflecting disparities in healthcare infrastructure, healthcare policies, and resource allocation. Although challenging, countries with efficient public health systems and strategic policies have demonstrated that the expansion of PD utilization can be achieved even in resource-limited settings [1,2]. Ensuring equitable access to RRT is essential for patient survival, as the underutilization of dialysis therapies has been associated with gaps in care and, ultimately, increased mortality among individuals with ESKD [3].

In Brazil, health is a constitutional right guaranteed by the State, which, in principle, ensures universal, comprehensive, and free healthcare to the population [4]. Established in 1988, the Brazilian Public Health System—known by its Portuguese acronym, SUS—ensures comprehensive, universal, and free access to healthcare for the entire population. SUS services range from primary medical care to complex treatments, including renal replacement therapy, and the provision of high-cost medications [5]. Despite progress over recent decades, SUS continues to face challenges, particularly in maintaining financial sustainability [6].

In Brazil, RRT options include kidney transplantation and dialysis, which comprises hemodialysis (HD), hemodiafiltration, and peritoneal dialysis. Approximately 80% of chronic dialysis procedures in the country are publicly funded by SUS. With 153,831 patients undergoing chronic dialysis in 2022, Brazil ranks third globally in absolute numbers of patients on dialysis, within a population of 203.08 million [7,8].

The SUS database (DATASUS) provides comprehensive data for reimbursement processes and epidemiological analyses [9]. However, there is a lack of studies detailing the clinical and epidemiological characteristics of patients undergoing RRT within the Brazilian Public Health System [10,11]. Such studies are not only scientifically relevant but also critically important for guiding policymakers and public health managers in developing nephrology-specific healthcare policies at a national level.

Over the past decade, the underfunding of RRT in Brazil has notably impacted the utilization of PD. In July 2010, the Brazilian Dialysis Census reported that 9.4% of chronic dialysis patients were on PD [12]. By 2022, this proportion had dropped to 4.7%, representing a 50% reduction [8]. Despite its advantages, such as reduced travel requirements for patients, PD has faced challenges including lower reimbursement rates compared to HD and inadequate training for nephrologists and healthcare personnel [13].

In response to these challenges, the last few years have seen initiatives aimed at promoting PD in Brazil. In 2024, the National Federation of Renal Patients, with the support of the Brazilian Society of Nephrology, released a manifesto advocating for the increased adoption of PD in Brazil [14]. The document outlines several key measures to enhance PD utilization, including the following. (1) Implementing Recommendation 005 of the National Health Council, which proposes expanding home-based PD in specialized chronic kidney disease (CKD) care services within SUS. (2) Supporting initiatives to expand PD offerings, with adequate funding for public and contracted private centers. (3) Encouraging existing centers to increase their capacity while fostering partnerships to train new centers. (4) Establishing reference centers for PD to serve patients across regions and provide professional training. (5) Ensuring clear patient information to facilitate informed decision-making in consultations with nephrologists.

In September 2024, representatives from National Societies of Nephrology across Latin American countries gathered in the Dominican Republic to discuss strategies for promoting PD in the region, resulting in the “*Declaración de Santo Domingo*” [15]. Similarly, advancing PD adoption has been established as a priority by the National Parliamentary Front for Nephrology in recent years, through discussions with the Ministry of Health. However, despite collective efforts, no effective public healthcare policies have been implemented to stimulate the adoption of PD in the country.

This study analyzes data from DATASUS to assess the evolution of PD utilization in the Brazilian Public Health System over a 10-year period (2014–2023), focusing on sociodemographic trends, clinical parameters, and regional variations. Understanding these trends and identifying barriers to PD expansion provide valuable insights into healthcare inequities at regional and national levels, inform resource allocation, and support the development of policies aimed at improving equitable access to this life-saving therapy.

## 2. Materials and Methods

### 2.1. Population

We conducted a retrospective cohort study using the SUS database (DATASUS) related to renal replacement therapy (RRT) procedures through dialysis, based on reimbursement data. The study included all prevalent and incident patients with chronic kidney disease (CKD) who underwent “RRT-related dialysis procedures” under the Brazilian Public Health System between January 2014 and December 2023. Ethical approval was waived due to the anonymized and public nature of the database.

### 2.2. Data Collection

The data were obtained from DATASUS—the health information system maintained by the Brazilian Ministry of Health, which provides information on the Brazilian Public Health System (SUS) [9]. Dialysis procedure data are accessible through the “outpatient procedure system” (SIA-ATD) [16], linked to reimbursement for RRT centers providing dialysis therapy for patients with chronic kidney failure. These centers are required to submit monthly reports. In the original dataset, records are organized by individual procedures, resulting in multiple entries for the same patient. To address this issue, we developed a computational algorithm to retrieve individual patient data. Using a unique identification code provided by SUS, we identified each case and reorganized procedures by patients. Ultimately, our electronic/mathematical system prevented duplicate records and consolidated procedures per patient, enabling inclusion in the final analysis dataset without risk of duplication. Patients with acute kidney injury are not included in the SIA-ATD and were therefore excluded from this study.

### 2.3. Clinical Variables

The variables studied included age, gender, ethnicity, Brazilian region, type of RRT (HD or PD), serology for HIV, hepatitis B, and hepatitis C. Additionally, we collected data on serum hemoglobin, phosphorus, intact parathyroid hormone (PTH), single-pool Kt/V (spKt/V), and serum albumin. Biochemical parameters were analyzed using the average of all available measures for each patient during the follow-up period. In general, biochemical tests—excluding PTH and serum albumin—were conducted monthly and documented on the same reimbursement form, as required by Brazilian regulations. PTH and serum albumin measurements were performed every three months. All dialysis centers utilized the intact PTH assay.

Ethnicity was self-reported and categorized as White, Brown/Black (mixed-race), Asian, and Indigenous. Brazilian regions were classified as North, Northeast, Central-West, Southeast, and South. The number of RRT centers was determined using the “National Register of Legal Entities” (CNPJ), a unique code assigned to each company in Brazil. To determine the number of cities providing RRT, we utilized codes from the Brazilian Institute of Geography and Statistics (IBGE) [17].

### 2.4. Adequacy Metrics

Monthly test results were classified within reference ranges defined by the Kidney Disease Outcomes Quality Initiative (KDOQI) guidelines [18]. Dialysis adequacy was assessed using spKt/V. The following parameters were considered within the target ranges: serum phosphorus between 3.5 and 5.5 mg/dL, intact PTH levels between 150 and 600 pg/mL, and hemoglobin levels between 10 and 12 g/dL.

### 2.5. Brazilian Epidemiological Data

We used data from IBGE for the total Brazilian population [19]. Population estimates from 2014 to 2021 were based on the 2010 census, while the 2022 data were derived from the Brazilian demographic census conducted that year. The 2022 census revealed that previous population estimates had been overestimated, leading to an underestimation of RRT prevalence rates for the years 2014 to 2021. The prevalence rate of RRT patients was calculated as the number of dialysis patients during the year divided by the Brazilian population on 1 July.

### 2.6. Outcomes

The outcomes analyzed included mortality, transfer to another RRT center, and recovery of renal function within one year among prevalent patients. We also assessed the incident survival of patients who started dialysis between January 2014 and December 2023.

### 2.7. Statistical Analysis

Descriptive epidemiological data were presented using medians and percentiles as measures of central tendency. Categorical variables were presented as numbers and frequencies. We assessed the distribution of the data using the Kolmogorov–Smirnov test and visual inspections, including histograms and Q-Q plots, to determine normality.

We applied Joinpoint regression analysis to estimate segmented log-linear trends over time. This method is particularly effective for analyzing time series data, identifying points of inflection in the population structure, and determining the timing of these changes [20]. For each identified segment, the Annual Percentage Change (APC) and its 95% confidence interval were calculated. Additionally, the Average Annual Percentage Change (AAPC) was determined to summarize the overall rate of change across the study period. The annual prevalence rate of dialysis therapy was calculated by dividing the total number of dialysis patients in a given year by the Brazilian population as of 1 July. The crude mortality rate was determined by dividing the number of deaths during the year by the total dialysis population in the same year. For survival analysis, we used the Kaplan–Meier method, with the start of dialysis therapy as the baseline and the study endpoint, date of death, or last follow-up date, whichever came first, as the outcome. All statistical analyses were performed using R software version 4.1.2.

## 3. Results

### 3.1. Socio-Demographic and Regional Characteristics

**Figure 1:**
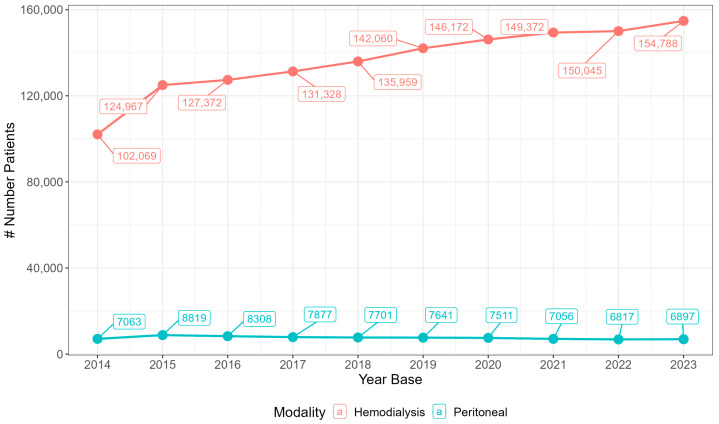
Number of patients undergoing RRT in Brazil within the Public Healthcare System (SUS) from 2014 to 2023, by modality (HD and PD).

**Figure 2:**
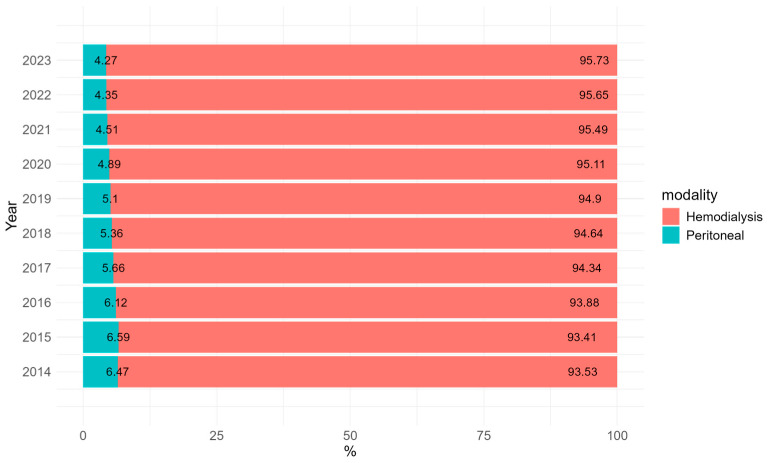
Percentage of patients undergoing RRT in Brazil within the Public Health System (SUS) from 2014 to 2023, by dialysis modality (hemodialysis and peritoneal dialysis).

Between 2014 and 2023, there was an increase in the number of patients undergoing HD, from 102,069 in 2014 to 154,788 in 2023. In contrast, the number of patients on PD decreased from 7063 in 2014 to 6897 in 2023. Proportionally, the percentage of PD in the total number of patients undergoing dialysis declined from 6.47% in 2014 to 4.27% in 2023 (Figure 1 and Figure 2, Table 1). The Brazilian regions with the highest proportion of patients undergoing RRT were the Southeast and Northeast regions (Table 1).

**Figure 3:**
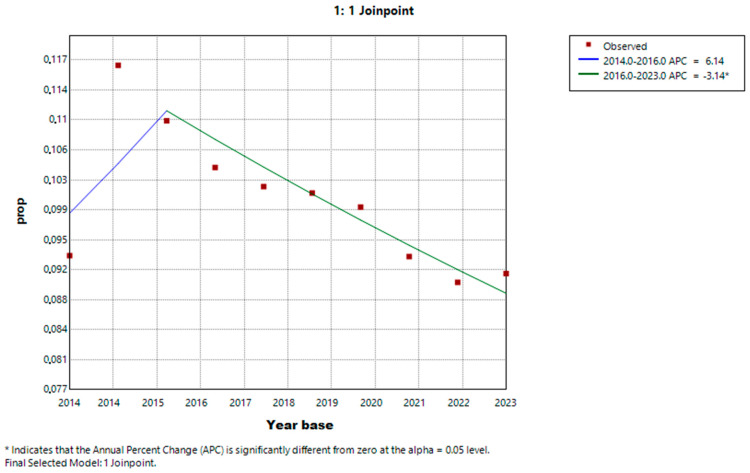
Joinpoint regression showing the variation in the proportion of PD over time in Brazil.

During the study period, there was an annual increase in the prevalence of HD, with an Adjusted Average Annual Percent Change (AAPC) of 4.42% [95% CI: 2.95%, 5.6%]. Conversely, PD showed an annual reduction, with an AAPC of −1.15% [95% CI: −2.82%, −0.02%], as shown in Table 2. Specifically for PD, there was a non-significant increase in frequency between 2014 and 2016, with an Annual Percent Change (APC) of 6.14% [95% CI: −1.84%, 12.40%], followed by a decline from 2016 to 2023, with an APC of −3.14% [95% CI: −8.41%, −1.47%], as illustrated in Figure 3.

When evaluating patients on PD during the study period, the median age increased from 58 years (45, 69) in 2014 to 59 years (45, 70) in 2023 (Table 3). During this interval, an annual increase in age was observed, with an AAPC of 0.132 [95% CI: 0.08, 0.17] (Table 4). The proportion of white patients decreased, with an AAPC of −2.36% [95% CI: −4.12, −0.59%], while the proportion of Brown/Black patients increased, with an AAPC of 1.84% [95% CI: 0.30, 3.33%].

**Figure 4:**
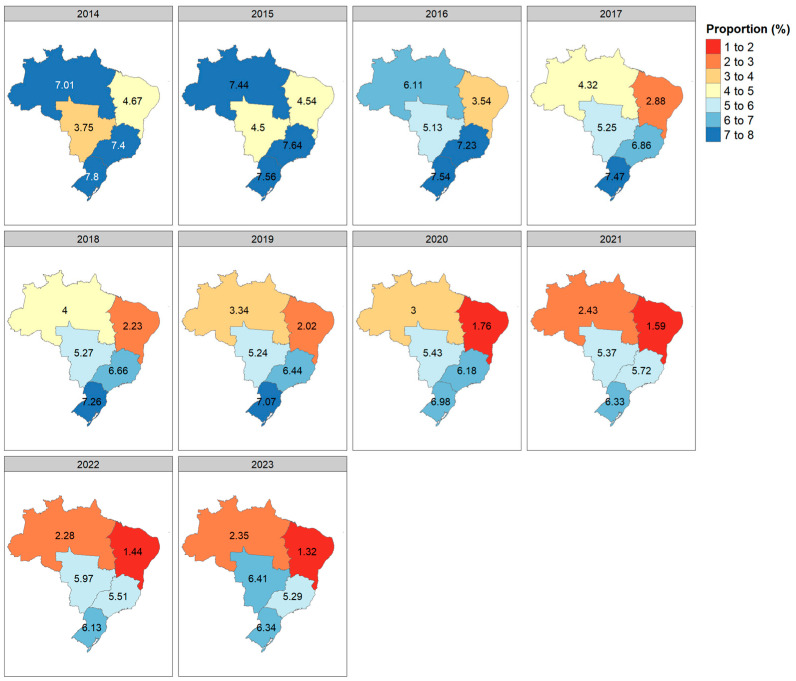
Proportion of patients undergoing peritoneal dialysis in Brazil’s Public Healthcare System (SUS) over time (2014–2023) by region (North, Northeast, Central-West, South, and Southeast). Values are expressed as percentages (%).

Regionally, there was an increase in the proportion of patients on PD in the Central-West region, with an AAPC of 9.68% [95% CI: 7.73, 11.51%]. In the Southeast and South regions, the proportion remained stable throughout the period (2014–2023); however, there was a reduction from 2016 to 2023 (Table 4). In the North and Northeast regions, there was a reduction, with AAPCs of −8.06% [95% CI: −12.18, −3.74%] and −9.86% [95% CI: −12.19, −7.55%], respectively. The distribution of patients by region over time is shown in Figure 4. Additionally, fewer patients initiated PD in recent years in the North and Northeast regions.

**Figure 5:**
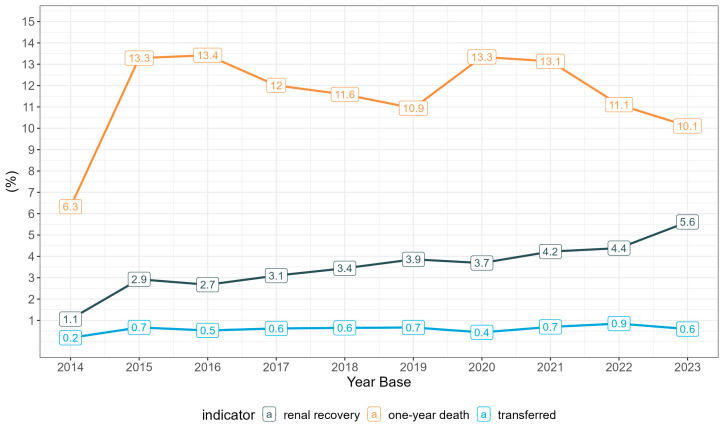
Proportion of events (renal function recovery, one-year mortality, and transfer between centers) among patients undergoing peritoneal dialysis in Brazil’s Public Healthcare System (SUS) over time (2014–2023). Values are expressed as percentages (%).

Additionally, there was a significant increase in the proportion of renal function recovery over time, with an AAPC of 14.88% [95% CI: 7.15, 19.65%] (Figure 5).

### 3.2. Laboratory Parameters

No significant changes were observed over time in laboratory test results, as shown in Table 5. The adequacy parameter ranges, according to the KDOQI guidelines, were consistent with international trends.

### 3.3. Prevalence

To calculate prevalence, the entire population undergoing RRT (HD and PD) was considered. There was an increase in prevalence over time, from 538 per million inhabitants in 2014 to 792 per million in 2023. The number of renal replacement therapy centers also increased, from 678 in 2014 to 716 in 2023. However, the number of centers offering PD decreased from 350 to 272 in the same period. The proportion of centers offering PD fell from 51.6% in 2014 to 37.9% in 2023 (Table 6).

### 3.4. Incident and Prevalent Cases

The number of prevalent and incident patients undergoing RRT and PD can be assessed in Table 7. The proportion of incident cases in RRT (HD and PD) varied from 23.3% to 28.1%, while the proportion of incident cases in PD ranged from 23.4% to 25.9%. Prevalence over time varied from 34.8 patients per million inhabitants in 2014 to 33.88 per million inhabitants in 2023.

### 3.5. Survival

**Figure 6:**
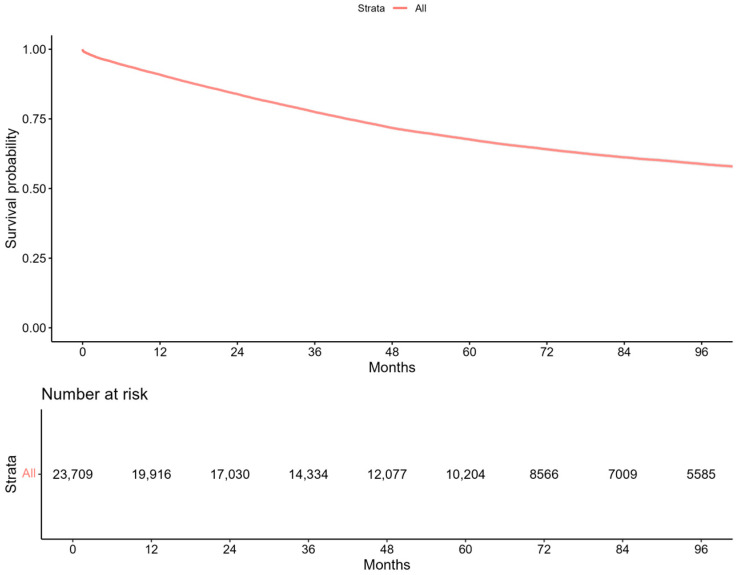
Survival on peritoneal dialysis in Brazil’s Public Healthcare System (SUS) from 2014 to 2023.

For survival analyses, unique patients during the study period were considered. Survival rates at 12, 24, 36, 60, and 96 months were as follows: 91% (90–91%), 84% (83–84%), 77% (77–78%), 68% (67–68%), and 59% (58–60%), as shown in Figure 6.

## 4. Discussions

In this study, patients undergoing RRT, with a focus on PD, were evaluated in Brazil’s Public Health System over a 10-year period. There was a reduction in PD utilization, decreasing from 6.5% in 2014 to 4.3% in 2023. A more pronounced decline was observed in the North and Northeast regions, whereas the Central-West region showed an increase, and the Southeast and South regions exhibited a decrease in PD prevalence between 2016 and 2023. The number of centers offering PD in Brazil dropped from 51.6% in 2014 to 37.9% in 2023. During the analyzed period, there was an increase in the mean age of patients and in the proportion of individuals of mixed-race (Brown/Black) ethnicity undergoing PD. Additionally, patients’ serum levels of hemoglobin, intact PTH, and phosphorus remained stable over time.

We used an innovative methodology to assess RRT patients in Brazil’s Public Health System, utilizing electronic reimbursement and mandatory registration data from the SUS. This approach was previously applied to analyze RRT in Brazil [21]. In 2014, national reimbursement records underwent modifications, leading to the inclusion of additional fields containing demographic, laboratorial, and epidemiological data not typically collected in SUS reimbursement records. This made the analysis of these datasets, such as SIA-ATD, particularly valuable for studies of this nature [16].

Significant variation in PD utilization has been observed worldwide, with approximately 11% of ESKD patients receiving this therapy. The greatest access challenges were identified in Africa and South Asia [22]. In the United States of America (USA), data from the USRDS indicated that around 8.3% of patients undergoing RRT, including those with kidney transplants, were on PD in 2022 [23]. Globally, PD is concentrated in a few countries—over 50% of PD patients worldwide are in China, the USA, Mexico, and Thailand [2,24]. In Brazil, PD utilization decreased from 6.5% in 2014 to 4.3% in 2023, corresponding to an annualized reduction rate of −1.15% [95% CI: −2.82, −0.02]. In 2013, data from the Brazilian Dialysis Census showed that approximately 9.2% of patients in Brazil were on PD [25,26]. Together with current data, a declining trend over the years is evident. Supporting this scenario, we observed a reduction in the number of dialysis centers offering PD (with at least one patient on PD), which dropped from 51.6% in 2014 to 37.9% in 2023. In our study, PD prevalence data also followed the same declining trend, from 34.8 to 33.8 per million population (pmp)—a figure below the estimated global PD prevalence of 38.1 pmp [22].

A key factor contributing to this decline may be the lower reimbursement rate for PD compared to HD, driven by insufficient public funding, which discourages its adoption by healthcare providers. Furthermore, inadequate training for healthcare personnel in PD management exacerbates the issue. Due to the low number of patients on PD, many nephrology trainees in Brazil, as in other countries, have limited exposure to this modality, reducing their confidence and ability to manage PD effectively [26]. The lack of effective public healthcare policies and adequate funding to incentivize RRT centers to expand or maintain PD programs are likely significant factors contributing to the declining number of facilities offering this treatment.

Similarly, low PD utilization in the USA is largely attributed to the lack of adequately trained medical professionals, both physicians and nurses. Many nephrology trainees have limited or no experience managing PD patients, reducing their confidence and ability to provide effective care in this modality [27]. In recent years, there has been an increasing effort to expand home dialysis in the USA, with the goal of achieving 80% of ESKD patients receiving treatment at home or undergoing kidney transplantation by 2025. This movement is driven by factors such as the USA’s lag in adopting home dialysis compared to other developed countries, the impact of conventional HD on patients’ quality of life, high treatment costs, and a shortage of nurses in dialysis units. PD is considered an effective solution, offering, in many cases, a less costly alternative with reduced transportation and human resource needs. However, despite significant growth in the incidence of new PD patients between 2010 and 2020, overall modality growth has been modest, increasing from 7.9% to 11.7% of dialysis patients due to losses from death, transplantation, and transfers to HD [27]. In low- and middle-income countries, PD adoption could improve access to treatment to meet growing demand, though challenges remain, such as lack of funding, infrastructure, and kidney health policies [24].

Brazil faces additional challenges due to its large territorial dimensions and regional inequalities. Some studies indicate that access to healthcare services is more unequal in the North and Northeast regions, likely due to insufficient infrastructure [28,29]. The Northeast region has the highest inequality, ranking last among the five regions in terms of Gross Regional Domestic Product (GRDP) per capita. This inequality can also be attributed to the limited healthcare infrastructure in this region [28]. In our study, the North region showed an annualized PD reduction of −8.06% [95% CI: −12.18, −3.74], while the Northeast recorded a reduction of −9.86% [95% CI: −12.19, −7.55]. Although no significant reduction was observed in the Southeast and South regions over the study period (2014–2023), there was an annualized reduction between 2016 and 2023 in both regions: −3.10% [95% CI: −7.65, −1.76] in the Southeast and −1.07% [95% CI: −9.2, 0.40] in the South.

Surprisingly, the Central-West region demonstrated both a relative and absolute increase in the number of PD patients. This trend could potentially be attributed to the dedicated efforts of certain dialysis centers and the positive influence of the Brazilian Federal District, which concentrates healthcare resources and is strategically located within the Central-West region. In recent years, the Brazilian Federal District faced a shortage of HD slots in the Public Health System for incident patients, a limitation that did not impact access to PD. Additionally, the lower logistical costs of delivering PD supplies to this region, compared to more distant areas such as the North and Northeast, might alleviate the financial burden—often a critical barrier—caused by the chronic underfunding of RRT in Brazil, making PD a more feasible option in the Central-West. These factors, though hypothetical, may help explain the observed increase in PD utilization in this region.

A geolocation study of dialysis units in Brazil showed that the distances traveled by patients were greater in the North region (average of 84.3 km) compared to the Southeast (average of 27.6 km). The proportion of patients traveling more than 40 km was also higher in the North (77%) compared to the Southeast (32%). The North and Central-West regions, which had the greatest travel distances, also have fewer dialysis centers and a vast geographic expanse [30]. Expanding PD in these regions could alleviate access difficulties, as this modality reduces the need for frequent travel—unlike in-center conventional HD, which requires travel to a dialysis center usually three times a week.

An effective alternative to increasing the use of PD in Brazil may be urgent-start PD, defined as the initiation of treatment within 14 days after the insertion of a Tenckhoff catheter. Urgent-start PD offers an alternative to HD in unplanned dialysis initiation scenarios, with outcomes comparable to planned dialysis. Furthermore, it can reduce complications and hospitalizations, facilitating the transition to outpatient treatment with PD, particularly for patients starting therapy in a hospital setting [31]. Studies conducted in Brazil have shown that urgent-start PD is safe and effective, even in patients not previously prepared for the therapy [32]. This approach can alleviate the pressure on HD, especially in regions where there is a shortage of HD slots [33,34].

In our study, we observed an increase in the median age of PD patients, rising from 58 (45–69) years in 2014 to 59 (45–70) years in 2023, representing an annualized rate of 0.132 [95% CI: 0.08, 0.17]. Similarly, for patients on RRT in Brazil, the median age increased from 57 years in 2015 to 59 years in 2023 [30]. These findings align with the rise in life expectancy in Brazil, which increased from 71.3 to 75.2 years between 2000 and 2017, accompanied by a more modest growth in healthy life expectancy, from 62.2 to 65.5 years [35].

The self-declared ethnic distribution among Brazilians, according to IBGE 2022, was as follows: Brown/Black (mixed-race) 55.5%, White 43.5%, and Asian/Indigenous 1% [36]. The racial distribution at the end of 2014 in this study was 47% White and 51% Brown/Black (mixed-race), similar to official national data from IBGE. There was an annualized increase of 1.84% [95% CI: 0.30, 3.33] in the Brown/Black (mixed-race) population, which may be explained by the reduction in the “not reported” category. In recent years, fewer patients were classified as “not reported”, and many were likely correctly categorized as mixed-race, partially accounting for the significant differences observed in our study over time.

We did not observe significant changes in laboratory parameters over time, with serum hemoglobin, intact PTH, and phosphorus levels remaining stable throughout the study period. The difficulty in meeting dialysis adequacy targets was consistent with findings from other studies on PD [37,38].

PD survival rates in our study at 12, 24, 36, 60, and 96 months were 91%, 84%, 77%, 68%, and 59%, respectively. These survival rates are similar to a single-center cohort study in Mexico, which reported 90% at 12 months, 89.7% at 24 months, and 83.2% at 36 months [39]. Generally, PD survival rates follow a consistent pattern across various studies. After 12 months, approximately 80–90% of patients are alive. This number drops to 70–80% after 24 months. By 48 months, survival rates decline to 50–65%, and long-term survival at 96 months is around 25–40%. Factors such as age, comorbidities, and complications like peritonitis can influence these outcomes [39,40]. The stability of survival rates observed in our study suggests that the quality of care for patients who remained on PD did not decline over time. However, reduced access to PD may disproportionately impact marginalized populations, likely increasing reliance on HD and introducing additional burdens, such as higher costs and logistical challenges. Policymakers must address these inequities by implementing targeted strategies to improve PD infrastructure, enhance reimbursement rates, and expand training programs to ensure equitable access to RRT nationwide.

Finally, the environmental sustainability of dialysis modalities has become a critical topic in the context of public health planning and the growing global focus on climate change mitigation. Green nephrology and ecodialysis committees have been established worldwide, including in Brazil. HD has been associated with substantial environmental impacts, including significant waste generation—over one million tons of disposable materials such as dialyzers and tubing—water consumption of approximately 500 L per session per patient, and energy usage estimated globally at more than two billion kWh annually. In contrast, although comprehensive studies evaluating the resource usage in the production of sterile fluids for PD are lacking, PD may offer certain advantages over HD in terms of reduced water usage and energy consumption [41].

This study had some limitations. We were unable to retrieve data on primary kidney disease or associated comorbidities, as many diagnoses were recorded using the generic ICD-10 code N18.9, indicating unspecified chronic kidney disease. Additionally, monthly laboratory test results for serum potassium were unavailable because this information is not included in dialysis reimbursement forms. Kt/V data were also not analyzed within KDOQI ranges due to the absence of residual kidney function data. The study did not evaluate peritonitis incidence or method failure rates, including conversion to HD. Lastly, data on the patients undergoing dialysis covered by private health insurance, which accounts for approximately 20% of RRT patients in Brazil, were not assessed, as they are not included in the public healthcare system database, DATASUS.

Despite these limitations, this study contributes to understanding the PD landscape within Brazil’s Public Health System over the past 10 years. There are still gaps to be addressed in identifying the underlying causes of the observed trends and exploring potential solutions. Future research should focus on longitudinal studies that evaluate the impact of specific public healthcare policies, such as ‘*PD First*’ initiatives, education programs, and financial incentives, on PD utilization in different countries or regions. Additionally, comparative studies examining the cost-effectiveness and patient outcomes of PD versus HD in diverse healthcare settings could provide valuable insights to guide policymaking and improve equitable access to PD worldwide.

## 5. Conclusions

This study highlighted a relative decline in PD utilization within the Public Health System in Brazil over a decade, with notable regional variations. The reduction in the number of centers offering this modality contributed to this downward trend. We identified a declining trend in PD utilization, particularly in the North and Northeast regions, and observed an increase in the age of patients undergoing treatment. Challenges to expanding PD use, including financial, technical, and regional issues, must be addressed to ensure equitable access to RRT in the country.

## Figures and Tables

**Table 1:** Number of patients undergoing RRT in Brazil within the Public Healthcare System (SUS) from 2014 to 2023, by dialysis modality and geographic region. The percentages in parentheses, listed below the absolute numbers, refer to the proportion within the total population on dialysis (HD and PD) in the Brazilian Public Health System.

	2014, n= 109,132	2015, n = 133,786	2016, n = 135,680	2017, n = 139,205	2018, n = 143,660	2019, n = 149,701	2020, n = 153,683	2021, n = 156,428	2022, n = 156,862	2023, n = 161,685
**Modality**										
HD	102,069 (94%)	124,967 (93%)	127,372 (94%)	131,328 (94%)	135,959 (95%)	142,060 (95%)	146,172 (95%)	149,372 (95%)	150,045 (96%)	154,788 (96%)
PD	7063 (6.5%)	8819 (6.6%)	8308 (6.1%)	7877 (5.7%)	7701 (5.4%)	7641 (5.1%)	7511 (4.9%)	7056 (4.5%)	6817 (4.3%)	6897 (4.3%)
**Region**										
Central-West	8427 (7.7%)	10,773 (8.1%)	11,287 (8.3%)	11,259 (8.1%)	12,096 (8.4%)	12,245 (8.2%)	12,442 (8.1%)	13,015 (8.3%)	12,492 (8.0%)	12,937 (8.0%)
Northeast	27,321 (25%)	33,353 (25%)	33,902 (25%)	35,958 (26%)	36,589 (25%)	39,244 (26%)	40,674 (26%)	41,236 (26%)	42,263 (27%)	44,389 (27%)
North	5276 (4.8%)	6811 (5.1%)	6707 (4.9%)	7152 (5.1%)	7624 (5.3%)	8286 (5.5%)	8620 (5.6%)	8758 (5.6%)	9420 (6.0%)	9488 (5.9%)
Southeast	52,530 (48%)	62,970 (47%)	63,895 (47%)	64,492 (46%)	66,373 (46%)	67,905 (45%)	69,493 (45%)	70,195 (45%)	69,778 (44%)	71,774 (44%)
South	15,578 (14%)	19,879 (15%)	19,889 (15%)	20,344 (15%)	20,978 (15%)	22,021 (15%)	22,454 (15%)	23,224 (15%)	22,909 (15%)	23,097 (14%)

**Table 2:** Annual Percent Growth Rate of changes in the frequency of peritoneal dialysis in Brazil’s Public Healthcare System over time (2014 to 2023).

Characteristic	Segment 1 +	APC (95% CI)	Segment 2 +	APC (95% CI)	AAPC (95% CI–Full Range)
**Modality**					
Hemodialysis	2014–2016	11.22 * [4.84, 17.8]	2016–2023	2.55 [−1.53, 3.63]	4.42 * [2.95, 5.60]
Peritoneal Dialysis	2014–2016	6.14 [−1.84, 12.40]	2016–2023	−3.14 * [−8.41, −1.47]	−1.15 * [−2.82, −0.02]

Annual Percentage Change (APC) and Average Annual Percentage Change (AAPC) estimates by Joinpoint Regression. * Indicates that AAPC or APC is significantly different from zero at the alpha = 0.05 level. + Segments in joinpoint regression represent periods with a consistent rate of change between identified joinpoints, indicating shifts in the trend.

**Table 3:** Characteristics of patients undergoing RRT through peritoneal dialysis in Brazil’s Public Healthcare System (SUS) from 2014 to 2023.

	2014, n = 7063	2015, n = 8819	2016, n = 8308	2017, n = 7877	2018, n = 7701	2019, n = 7641	2020, n = 7511	2021, n = 7056	2022, n = 6817	2023, n = 6897
**Age (years)**	58 (45, 69)	58 (45, 69)	58 (45, 69)	59 (45, 69)	59 (45, 69)	59 (45, 69)	59 (45, 70)	59 (45, 69)	59 (45, 69)	59 (45, 70)
Less than 18 years	327 (4.6%)	446 (5.1%)	372 (4.5%)	334 (4.2%)	328 (4.3%)	362 (4.7%)	357 (4.8%)	295 (4.2%)	291 (4.3%)	269 (3.9%)
More than 60 years	3323 (47%)	4207 (48%)	3919 (47%)	3773 (48%)	3667 (48%)	3706 (49%)	3700 (49%)	3482 (49%)	3320 (49%)	3423 (50%)
**Gender**										
Female	3802 (54%)	4726 (54%)	4468 (54%)	4164 (53%)	3984 (52%)	3982 (52%)	3907 (52%)	3681 (52%)	3554 (52%)	3606 (52%)
Male	3261 (46%)	4093 (46%)	3840 (46%)	3713 (47%)	3717 (48%)	3659 (48%)	3604 (48%)	3375 (48%)	3263 (48%)	3291 (48%)
**Ethnicity**										
Yellow/ Indigenous	126 (1.8%)	158 (1.8%)	144 (1.7%)	138 (1.8%)	100 (1.3%)	75 (1.0%)	82 (1.1%)	89 (1.3%)	157 (2.3%)	155 (2.2%)
Caucasian/White	3351 (47%)	4148 (47%)	3877 (47%)	3610 (46%)	3559 (46%)	3438 (45%)	3224 (43%)	3037 (43%)	3096 (45%)	3240 (47%)
Not Informed	976 (14%)	1239 (14%)	1255 (15%)	1171 (15%)	1020 (13%)	997 (13%)	963 (13%)	840 (12%)	298 (4.4%)	6 (<0.1%)
Brown/Black	2610 (37%)	3274 (37%)	3032 (36%)	2958 (38%)	3022 (39%)	3131 (41%)	3242 (43%)	3090 (44%)	3266 (48%)	3496 (51%)
**Region**										
Central-West	316 (4.5%)	485 (5.5%)	579 (7.0%)	591 (7.5%)	637 (8.3%)	642 (8.4%)	675 (9.0%)	699 (9.9%)	746 (11%)	829 (12%)
Northeast	1276 (18%)	1514 (17%)	1199 (14%)	1034 (13%)	815 (11%)	792 (10%)	714 (9.5%)	655 (9.3%)	610 (8.9%)	584 (8.5%)
North	370 (5.2%)	507 (5.7%)	410 (4.9%)	309 (3.9%)	305 (4.0%)	277 (3.6%)	259 (3.4%)	213 (3.0%)	215 (3.2%)	223 (3.2%)
Southeast	3886 (55%)	4811 (55%)	4621 (56%)	4424 (56%)	4420 (57%)	4373 (57%)	4295 (57%)	4018 (57%)	3842 (56%)	3797 (55%)
South	1215 (17%)	1502 (17%)	1499 (18%)	1519 (19%)	1524 (20%)	1557 (20%)	1568 (21%)	1471 (21%)	1404 (21%)	1464 (21%)
**Positive serology** **for HIV**	40 (0.6%)	89 (1.0%)	55 (0.7%)	54 (0.7%)	82 (1.1%)	148 (1.9%)	144 (1.9%)	175 (2.5%)	191 (2.8%)	75 (1.1%)
**Positive serology** **for Hepatitis B**	28 (0.4%)	91 (1.0%)	38 (0.5%)	45 (0.6%)	64 (0.8%)	156 (2.0%)	124 (1.7%)	99 (1.4%)	184 (2.7%)	63 (0.9%)
**Positive serology** **for Hepatitis C**	108 (1.5%)	185 (2.1%)	148 (1.8%)	128 (1.6%)	131 (1.7%)	172 (2.3%)	184 (2.4%)	205 (2.9%)	219 (3.2%)	89 (1.3%)
**Hospital Discharge**	75 (1.1%)	258 (2.9%)	222 (2.7%)	244 (3.1%)	265 (3.4%)	295 (3.9%)	277 (3.7%)	298 (4.2%)	299 (4.4%)	387 (5.6%)
**Transfer**	13 (0.2%)	59 (0.7%)	44 (0.5%)	49 (0.6%)	50 (0.6%)	51 (0.7%)	33 (0.4%)	49 (0.7%)	58 (0.9%)	41 (0.6%)
**Deaths**	447 (6.3%)	1172 (13%)	1115 (13%)	946 (12%)	892 (12%)	836 (11%)	1002 (13%)	927 (13%)	757 (11%)	697 (10%)

Categorical variables expressed as numbers and percentages; numerical variables expressed as medians and percentiles (25th and 75th).

**Table 4:** Annual Percent Growth Rate of the characteristics of patients undergoing peritoneal dialysis in Brazil’s Public Healthcare System (SUS) over time, from 2014 to 2023.

Characteristic	Segment 1 +	APC (95% CI)	Segment 2 +	APC (95% CI)	AAPC (95% CI–Full Range)
**Age (years)**	2014–2023	0.132 * [0.08, 0.17]			0.132 * [0.08, 0.17]
Less than 18	2014–2023	−3.35 * [−5.99, −0.71]			−3.35 * [−5.99, −0.71]
More than 60	2014–2016	5.91 [−1.41, 10.75]	2016–2023	−3.72 * [−7.88, −2.44]	−1.66 * [−3.11, −0.77]
**Gender**					
Female	2014–2016	7.21 [−1.36, 15.2]	2016–2023	−2.81 * [−8.91, −1.01]	−0.66 [−2.57, 0.76]
Male	2014–2016	5.19 [−3.08, 15.56]	2016–2023	−3.43 * [−9.71, −0.33]	−1.58 * [−3.38, −0.24]
**Ethnicity**					
Yellow/Indigenous	2014–2020	−10.91 * [−36.1, −0.43]	2020–2023	27.9 [−1.89, 85.87]	0.49 [−7.37, 6.77]
Caucasian/White	2014–2023	−2.36 * [−4.12, −0.59]			−2.36 * [−4.12, −0.59]
Not Informed	2014–2021	0.53 [−14.29, 22.95]	2021–2023	−90.62 * [−95.1, −76.2]	−40.65 * [−47.8, −31.4]
Brown/Black	2014–2023	1.84 * [0.30, 3.33]			1.84 * [0.30, 3.33]
**Brazilian Region**					
Central-West	2014–2016	31.35 * [17.7, 42.72]	2016–2023	4.18 * [1.27, 6.01]	9.68 * [7.73, 11.51]
Northeast	2014–2023	−9.86 * [−12.19, −7.55]			−9.86 * [−12.19, −7.55]
North	2014–2023	−8.06 * [−12.18, −3.74]			−8.06 * [−12.18, −3.74]
Southeast	2014–2016	7.09 [−0.14, 15.28]	2016–2023	−3.10 * [−7.65, −1.76]	−0.76 [−2.44, 0.55]
South	2014–2016	11.23 * [1.95, 22.96]	2016–2023	−1.07 [−9.2, 0.40]	1.53 [−0.92, 3.47]
**Positive serology for HIV**	2014–2021	23.13 * [7.67, 76.13]	2021–2023	−29.71 [−54.8, 12.9]	8.71 [−0.25, 20.46]
**Positive serology for Hepatitis B**	2014–2023	13.53 [−5.48, 35.90]			13.53 [−5.48, 35.90]
**Positive serology for Hepatitis C**	2014–2021	7.76 [−8.35, 51.00]	2021–2023	−27.5 [−51.3, 7.65]	−1.32 [−8.63, 8.22]
**Recovery of renal function**	2014–2016	61.8 * [15.49, 98.5]	2016–2023	4.17 [−15.94, 10.9]	14.88 * [7.15, 19.65]
**Patients transferred between centers**	2016–2016	72.29 * [4.68, 156]	2016–2023	−2.82 [−33.5, 9.29]	10.36 [−1.18, 19.3]
**One-year mortality**	2014–2016	44.9 * [3.8, 95.1]	2016–2023	−6.32 * [−25.6, −0.08]	3.2 [−4.60, 9.49]

Annual Percentage Change (APC) and Average Annual Percentage Change (AAPC) estimates by Joinpoint Regression. * Indicates that AAPC or APC is significantly different from zero at the alpha = 0.05 level. + Segments in joinpoint regression represent periods with a consistent rate of change between identified joinpoints, indicating shifts in the trend.

**Table 5:** Laboratory parameters of patients undergoing peritoneal dialysis in Brazil’s Public Healthcare System (SUS) from 2014 to 2023.

	2014, n = 7063	2015, n = 8819	2016, n = 8308	2017, n = 7877	2018, n = 7701	2019, n = 7641	2020, n = 7511	2021, n = 7056	2022, n = 6817	2023, n = 6897
**Hemoglobine (g/dL)**	11.24 (10.00, 12.20)	11.00 (9.63, 12.08)	11.00 (9.80, 12.08)	10.92 (9.83, 12.00)	10.33 (8.75, 11.61)	10.78 (9.50, 11.86)	10.83 (9.71, 12.00)	10.93 (9.67, 11.86)	10.68 (9.26, 11.80)	10.83 (9.66, 11.79)
**Phosphorus (mg/dL)**	4.60 (3.60, 6.00)	4.77 (3.75, 6.00)	4.67 (3.75, 5.92)	4.82 (3.83, 5.92)	5.00 (3.70, 6.08)	4.97 (3.85, 6.00)	5.00 (4.08, 6.25)	5.00 (4.00, 6.17)	4.80 (3.91, 5.95)	5.00 (4.00, 6.00)
**PTH (pg/mL)**	257 (113, 512)	300 (135, 573)	288 (129, 554)	283 (138, 535)	287 (132, 535)	316 (151, 624)	345 (168, 710)	330 (161, 688)	320 (148, 676)	326 (157, 615)
**spKt/V**	1.00 (1.00, 1.50)	1.00 (0.92, 1.30)	1.00 (1.00, 1.20)	1.00 (1.00, 1.20)	1.00 (0.67, 1.20)	1.00 (0.89, 1.21)	1.00 (1.00, 1.72)	1.00 (0.93, 1.44)	1.13 (0.78, 1.49)	1.05 (0.67, 1.48)
**Albumine (g/dL)**	3.32 (3.00, 4.00)	3.33 (2.75, 3.93)	3.25 (2.63, 4.00)	3.25 (2.75, 4.00)	3.30 (2.74, 4.00)	3.50 (3.00, 4.00)	3.57 (3.00, 4.00)	3.60 (3.00, 4.00)	3.39 (2.80, 3.83)	3.55 (3.00, 3.90)

PTH: intact parathyroid hormone; spKt/V (single pool Kt/V—dialysis adequacy parameter); Hb: hemoglobin; values are expressed as medians and percentiles (25th and 75th).

**Table 6:** Brazilian population, number and percentage of dialysis centers offering peritoneal dialysis, total number of patients, and prevalence rate of dialysis-related RRT in Brazil’s Public Healthcare System (SUS) from 2014 to 2023.

Year	RRT Prevalence (n)	Population +	RRT Prevalence pmp *	Total dialysis Centers (n)/Centers Offering PD	Proportion of Centers Offering PD	Cities with Dialysis (n)/Cities with PD
2014	109,132	202,768,562 +	538	678/350	51.6%	405/220
2015	133,786	204,450,049 +	654	692/363	52.4%	407/227
2016	135,680	206,081,432 +	658	687/344	50.1%	407/223
2017	139,205	207,660,929 +	670	693/338	48.7%	412/219
2018	143,660	208,494,900 +	689	702/325	46.3%	419/213
2019	149,701	210,147,125 +	712	713/318	44.6%	425/211
2020	153,683	211,755,692 +	725	714/311	43.5%	425/209
2021	156,428	213,317,639 +	733	723/298	41.2%	430/202
2022	156,862	203,080,756 &	772	724/283	39.1%	442/198
2023	161,685	204,136,776 ++	792	716/272	37.9%	449/191

RRT: Renal replacement therapy. pmp: per million population * Prevalence rate of dialysis patients per million population. + Brazilian population estimate https://sidra.ibge.gov.br/tabela/6579#resultado (accessed on 2 August 2024); & 2022 Brazilian population census; ++ population estimate derived from the 2022 census.

**Table 7:** Incident and prevalent patients undergoing dialysis-related RRT in the Public Healthcare System (SUS) from 2014 to 2023.

Year	Prevalents in RRT	Incidents in RRT	% of Incidents in RRT	Prevalents in PD	Incidents in PD	% of Incidents in PD	PD Prevalence pmp
**2014**	109,132	25,452	23.3%	7063	1834	25.9%	34.8
**2015**	133,786	37,065	27.7%	8819	2277	25.8%	43.1
**2016**	135,680	36,692	27.1%	8308	2122	25.5%	40.3
**2017**	139,205	36,663	26.3%	7877	1962	24.9%	37.9
**2018**	143,660	37,613	26.2%	7701	1872	24.3%	36.9
**2019**	149,701	39,841	26.6%	7641	1870	24.4%	36.4
**2020**	153,683	39,173	25.5%	7511	1798	23.9%	35.5
**2021**	156,428	43,987	28.1%	7056	1808	25.6%	33.1
**2022**	156,862	42,253	26.9%	6817	1598	23.4%	33.6
**2023**	161,685	43,887	27.1%	6897	1774	25.7%	33.8

RRT: Renal replacement therapy. pmp: per million population.

## Data Availability

All the data are available from the corresponding author upon reasonable request.
